# Supplementary material for: A decomposition of light’s spin angular momentum density
Source: Light Sci Appl. 2024 Jul 10;13:160. doi: 10.1038/s41377-024-01447-9 (PMC11237040; doi:10.1038/s41377-024-01447-9)
Supplement: Supplementary file 1 — Supplementary Information [file 41377_2024_1447_MOESM1_ESM.pdf]

Supplementary Information for 'A decomposition of light's spin angular momentum density'

Alex J. Vernon[1,2], Sebastian Golat[1,2], Claire Rigouzzo[1], Eugene A. Lim[1], and Francisco J. Rodríguez-Fortuño*[1,2]

[1]Department of Physics, King's College London, Strand, London WC2R 2LS, UK
[2]London Centre for Nanotechnology

This document supports the main text with four main components. In section 1, we provide an alternative version of Table I of the manuscript, expressed completely using index notation, which highlights the similarities and differences between vector and tensor waves. Section 2 clarifies an expression presented by the authors of [1]. Next, section 3 is a discussion of the equivalent SAM density decomposition in linerised acoustics, which informs the first column of Table II of the manuscript. In section 4, details of the units of the quantities handled in the manuscript's 4-vector decomposition are given. Finally in section 5, the electromagnetic spin decomposition is presented in a general gauge, supporting comments made in the in 4-vector section of the main text.

*francisco.rodriguez_fortuno@kcl.ac.uk

1 Alternative table (index notation)

An alternative version of Table 1 from the manuscript is given below, where all quantities are expressed using index notation.

	**Linearised acoustics**	**Electromagnetism**	**Linearised gravity**
Potential field	Scalar field φ	Vector field A^μ	Tensor field $h_{\mu\nu}$
Choice of gauge	n/a	Coulomb gauge: $A^0 = 0$ $\partial_i A^i = 0$	Transverse traceless gauge: $h_{0\mu} = h^i{}_i = 0$ $\partial_i h^{ij} = 0$
Helmholtz equation	$\nabla^2 \varphi = -k^2 \varphi$	$\nabla^2 A^i = -k^2 A^i$	$\nabla^2 h^{ij} = -k^2 h^{ij}$
Fields	$P = -i\omega\rho\varphi$ $v^i = \partial^i \varphi$	$E^i = i\omega A^i$ $\mu_0 H^i = \epsilon^{ijk}\partial_j A_k$	$E^{ij} = i\omega h^{ij}$ $\mu_0 H^{ij} = \epsilon^{ikm}\partial_k h^j{}_m$
Spin	$\frac{\rho}{2\omega}\epsilon^{ijk}\mathrm{Im}\{v_j^* v_k\}$	$\frac{1}{4\omega}\epsilon^{ijk}\mathrm{Im}\{\epsilon_0 E_j^* E_k + \mu_0 H_j^* H_k\}$	$\frac{1}{2\omega}\epsilon^{ijk}\mathrm{Im}\{\epsilon_0 E_{jl}^* E_k{}^l + \mu_0 H_{jl}^* H_k{}^l\}$
Canonical spin	0	$\frac{1}{4\omega^2}\mathrm{Re}\{E_j^*\partial_i H^j - H_j^*\partial_i E^j\}$	$\frac{1}{2\omega^2}\mathrm{Re}\{E_{jn}^*\partial_i H^{jn} - H_{jn}^*\partial_i E^{jn}\}$
Poynting spin	$\frac{1}{2\omega^2}\epsilon^{ijk}\partial_j \frac{1}{2}\mathrm{Re}\{P^* v_k\}$	$\frac{1}{2\omega^2}\epsilon^{ijk}\partial_j \frac{1}{2}\mathrm{Re}\{\epsilon_{klm} E^{*l} H^m\}$	$\frac{1}{\omega^2}\varepsilon^{ijk}\partial_j \frac{1}{2}\mathrm{Re}\{\varepsilon_{klm} E^{*l}{}_n H^{mn}\}$
Energy density	$\frac{1}{4}\{\beta p^* p + \rho v_i^* v^i\}$	$\frac{1}{4}\{\epsilon_0 E_j^* E^j + \mu_0 H_j^* H^j\}$	$\frac{1}{4}\{\epsilon_0 E_{jk}^* E^{jk} + \mu_0 H_{jk}^* H^{jk}\}$
Maxwell(-like) equations	$\partial_i P = i\omega\rho v^i$ $\partial_i v^i = i\omega\beta P$ $\epsilon^{ijk}\partial_j v_k = 0$	$\partial_i E^i = 0$ $\partial_i H^i = 0$ $\epsilon^{ijk}\partial_j E_k = i\omega\mu_0 H^i$ $\epsilon^{ijk}\partial_j H_k = -i\omega\epsilon_0 E^i$	$\partial_i E^{ij} = 0$ $\partial_i H^{ij} = 0$ $\epsilon^{ijk}\partial_j E_k{}^l = i\omega\mu_0 H^{il}$ $\epsilon^{ijk}\partial_j H_k{}^l = -i\omega\epsilon_0 E^{il}$

Table 1: Comparison between acoustics, electromagnetism and linearised gravity. Table inspired by [2–4]. The parameter $\epsilon_0 = 1/(c^2\mu_0) = c^2/(32\pi G)$ for linearised gravity was chosen such that the time-averaged energy density takes the same form as for electromagnetism [5].

2 Expression for spin by Shi et. al

The authors of [1] provided an expression of total SAM density $\mathbf{S}$ decomposed in to two terms (see Eq. (1) of the cited work), which we reprint here,

$$\mathbf{S} = \underbrace{\frac{1}{4\omega^2}\mathrm{Re}\{-(\nabla\otimes\mathbf{E}^*)\cdot\mathbf{H} - (\nabla\otimes\mathbf{E})^T\cdot\mathbf{H}^* + (\nabla\otimes\mathbf{H}^*)\cdot\mathbf{E} + (\nabla\otimes\mathbf{H})^T\cdot\mathbf{E}^*\}}_{\text{1st term} \equiv \mathbf{s}_c - \mathbf{s}_p} + \underbrace{\frac{1}{\omega^2}\nabla\times\mathbf{P}}_{\text{2nd term} \equiv 2\mathbf{s}_p} \quad . \tag{S1}$$

In their notation, $\nabla \otimes \mathbf{A}$ is a 3×3 matrix whose elements are $(\nabla \otimes \mathbf{A})_{ij} = \nabla_j A_i$. Here, we would like to align our expression Eq. (4) of the main text with Shi et. al's expression. Equation (S1) is different from Eq. (4) of the main text; the first term is much longer and the second term, proportional to the curl of the Poynting vector, is in fact a factor of two larger than our definition of Poynting spin ($= 2\mathbf{s}_p$). This is because, as the underbraces suggest, the first term is equal to $\mathbf{s}_c - \mathbf{s}_p$ where $\mathbf{s}_c$ and $\mathbf{s}_p$ are canonical and Poynting spins as we defined them in the article. We may show this by separating the first term into two chunks and expanding the matrix-vector multiplication. For the first chunk:

$$\frac{1}{4\omega^2}\mathrm{Re}\{-(\nabla \otimes \mathbf{E})^T \cdot \mathbf{H}^* + (\nabla \otimes \mathbf{H})^T \cdot \mathbf{E}^*\} =$$

$$\frac{1}{4\omega^2}\mathrm{Re}\left\{ -\begin{pmatrix} \frac{\partial E_x}{\partial x} & \frac{\partial E_y}{\partial x} & \frac{\partial E_z}{\partial x} \\ \frac{\partial E_x}{\partial y} & \frac{\partial E_y}{\partial y} & \frac{\partial E_z}{\partial y} \\ \frac{\partial E_x}{\partial z} & \frac{\partial E_y}{\partial z} & \frac{\partial E_z}{\partial z} \end{pmatrix} \begin{pmatrix} H_x^* \\ H_y^* \\ H_z^* \end{pmatrix} + \begin{pmatrix} \frac{\partial H_x}{\partial x} & \frac{\partial H_y}{\partial x} & \frac{\partial H_z}{\partial x} \\ \frac{\partial H_x}{\partial y} & \frac{\partial H_y}{\partial y} & \frac{\partial H_z}{\partial y} \\ \frac{\partial H_x}{\partial z} & \frac{\partial H_y}{\partial z} & \frac{\partial H_z}{\partial z} \end{pmatrix} \begin{pmatrix} E_x^* \\ E_y^* \\ E_z^* \end{pmatrix} \right\} \tag{S2}$$

$$= \frac{1}{4\omega^2}\mathrm{Re}\{-H_x^*\nabla E_x - H_y^*\nabla E_y - H_z^*\nabla E_z + E_x^*\nabla H_x + E_y^*\nabla H_y + E_z^*\nabla H_z\}$$

$$= \frac{1}{4\omega^2}\mathrm{Re}\{\mathbf{E}^* \cdot (\nabla)\mathbf{H} - \mathbf{H}^* \cdot (\nabla)\mathbf{E}\} = \mathbf{s}_c.$$

And expanding the second, we have,

$$\frac{1}{4\omega^2}\mathrm{Re}\{-(\nabla \otimes \mathbf{E}^*) \cdot \mathbf{H} + (\nabla \otimes \mathbf{H}^*) \cdot \mathbf{E}\} =$$

$$\frac{1}{4\omega^2}\mathrm{Re}\left\{ -\begin{pmatrix} \frac{\partial E_x^*}{\partial x} & \frac{\partial E_x^*}{\partial y} & \frac{\partial E_x^*}{\partial z} \\ \frac{\partial E_y^*}{\partial x} & \frac{\partial E_y^*}{\partial y} & \frac{\partial E_y^*}{\partial z} \\ \frac{\partial E_z^*}{\partial x} & \frac{\partial E_z^*}{\partial y} & \frac{\partial E_z^*}{\partial z} \end{pmatrix} \begin{pmatrix} H_x \\ H_y \\ H_z \end{pmatrix} + \begin{pmatrix} \frac{\partial H_x^*}{\partial x} & \frac{\partial H_x^*}{\partial y} & \frac{\partial H_x^*}{\partial z} \\ \frac{\partial H_y^*}{\partial x} & \frac{\partial H_y^*}{\partial y} & \frac{\partial H_y^*}{\partial z} \\ \frac{\partial H_z^*}{\partial x} & \frac{\partial H_z^*}{\partial y} & \frac{\partial H_z^*}{\partial z} \end{pmatrix} \begin{pmatrix} E_x \\ E_y \\ E_z \end{pmatrix} \right\} \tag{S3}$$

$$= \frac{1}{4\omega^2}\mathrm{Re}\{-H_x\partial_x\mathbf{E}^* - H_y\partial_y\mathbf{E}^* - H_z\partial_z\mathbf{E}^* + E_x\partial_x\mathbf{H}^* + E_y\partial_y\mathbf{H}^* + E_z\partial_z\mathbf{H}^*\}$$

$$= \frac{1}{4\omega^2}\mathrm{Re}\{-(\mathbf{H} \cdot \nabla)\mathbf{E}^* + (\mathbf{E}^* \cdot \nabla)\mathbf{H}\} = \frac{1}{4\omega^2}\mathrm{Re}\{-\nabla \times (\mathbf{E}^* \times \mathbf{H})\} = -\mathbf{s}_p.$$

where we have used the identity $\nabla \times (\mathbf{a} \times \mathbf{b}) = \mathbf{a}(\nabla \cdot \mathbf{b}) - \mathbf{b}(\nabla \cdot \mathbf{a}) + (\mathbf{b} \cdot \nabla)\mathbf{a} - (\mathbf{a} \cdot \nabla)\mathbf{b}$ and Gauss' law in free space, and the fact that $\mathrm{Re}\{\mathbf{a}^* \cdot \mathbf{b}\} = \mathrm{Re}\{\mathbf{a} \cdot \mathbf{b}^*\}$. Combining the above two expansions, we confirm that Eq. (S1) is equivalent to $\mathbf{S} = \mathbf{s}_c - \mathbf{s}_p + 2\mathbf{s}_p = \mathbf{s}_c + \mathbf{s}_p$.

3 Decomposition in linearised acoustics

An acoustic wave field can be described by a scalar pressure field P and a vector velocity field $\mathbf{v}$ which, in linearised acoustic theory, share a Maxwell-like relation [2, 6]. The time-harmonic equations are,

$$\nabla \cdot \mathbf{v} = i\beta\omega P, \tag{S4}$$

$$\nabla P = i\rho\omega\mathbf{v}. \tag{S5}$$

where the constants β and ρ are the acoustic medium's compressibility and mass density respectively. Compared to photons, acoustic phonons, which are spin-0 quanta in this regime, do not give acoustic fields as rich a vector structure as light. Constraining the velocity vector is the longidutinality condition, that is $\nabla \times \mathbf{v} = \mathbf{0}$, a more restrictive analogy to light's transversality condition due to Gauss' law. Yet, $\mathbf{v}$ can still rotate and generate acoustic SAM, which is expressed (time-averaged) by,

$$\mathbf{S}_{ac} = \frac{\rho}{2\omega}\mathrm{Im}\{\mathbf{v}^* \times \mathbf{v}\}. \tag{S6}$$

In time-harmonic acoustic fields, where the velocity $\mathbf{v}$ and displacement field $\mathbf{r}$ vectors are related by $\mathbf{v} = -i\omega\mathbf{r}$, particles in the acoustic medium have an elliptical motion. Meanwhile, an acoustic analogy to the Poynting vector can be defined by mixing the acoustic pressure and velocity fields,

$$\mathbf{P}_{ac} = \frac{1}{2}\mathrm{Re}\{P^*\mathbf{v}\}. \tag{S7}$$

Taking the curl of $\mathbf{P}_{ac}$ and following with use of Eq. (S5), the acoustic SAM emerges in what appears to be the acoustic analogy to the presented electromagnetic spin decomposition:

$$\mathbf{S}_{ac} = -\frac{1}{2\omega^2}\mathrm{Re}\{P^*(\nabla \times \mathbf{v})\} + \frac{1}{2\omega^2}\nabla \times \mathbf{P}_{ac}. \tag{S8}$$

Due to the longitudinality condition $\nabla \times \mathbf{v} = \mathbf{0}$, the first term in Eq. (S8) vanishes leaving the acoustic spin entirely proportional to the curl of the acoustic Poynting vector. Transverse phonons for which $\nabla \times \mathbf{v} \neq \mathbf{0}$ can occur in viscous fluids or solids, although in these media, the acoustic field could no longer be described by a linearised theory. Compared to the electromagnetic spin decompostion, however, Eq. (S8) highlights the structural distinction between spin-0 and spin-1 fields as only vorticity in the flow of energy can generate SAM in a linear acoustic field.

4 On units

Discussions in this section are related to the 4-vector extension of the decomposition presented in section 4.2 of the manuscript. The field strength tensor $F_{\mu\nu}$ and conjugate field strength tensor $G_{\mu\nu}$ are not of the same units. The former has SI units $\mathrm{kg\,s^{-4}\,A^{-1}}$ whilst the latter has SI units $\mathrm{m^{-1}\,kg\,s^{-5}\,A^{-1}}$. This comes from the form of the conjugate field strength tensor that can be derived using the companion Mathematica notebook [7]:

$$G_{\mu\nu} = \begin{bmatrix} 0 & H_x/c & H_y/c & H_z/c \\ -H_x/c & 0 & -\epsilon_0 E_z & \epsilon_0 E_y \\ -H_y/c & \epsilon_0 E_z & 0 & -\epsilon_0 E_x \\ -H_z/c & -\epsilon_0 E_y & H_x/c^2 & 0 \end{bmatrix}. \tag{S9}$$

Finally, we summarize all the SI units of the mentioned tensor and constants:

	$\mathbf{E}$	$\mathbf{H}$	ϵ_0	μ_0	$\mathbf{A}$	ϕ	$\mathbf{C}$
Units	$\mathrm{m\,kg\,s^{-3}\,A^{-1}}$	$\mathrm{A\,m^{-1}}$	$\mathrm{kg^{-1}\,m^{-3}\,s^4\,A^{-2}}$	$\mathrm{kg\,m\,s^{-2}\,A^{-2}}$	$\mathrm{kg\,m\,s^{-2}\,A^{-1}}$	$\mathrm{kg\,m^2\,s^{-3}\,A^{-1}}$	$\mathrm{A\,m^{-1}\,s}$

Table 2: Summary of the SI units of every tensors and constants mentioned in this work

5 On the choice of gauge

The electric field and magnetic field, respectively $\mathbf{E}$ and $\mathbf{H}$, can be expressed in terms of a scalar potential ϕ and a vector potential $\mathbf{A}$,

$$\begin{aligned} \boldsymbol{\mathcal{E}} &= -\frac{\partial \boldsymbol{\mathcal{A}}}{\partial t} - \nabla\varphi \\ \mu_0 \boldsymbol{\mathcal{H}} &= \nabla \times \boldsymbol{\mathcal{A}} \end{aligned} \tag{S10}$$

and respectively for the conjugate vector potential:

$$\begin{aligned} \epsilon_0 \boldsymbol{\mathcal{E}} &= -\nabla \times \boldsymbol{\mathcal{C}} \\ \boldsymbol{\mathcal{H}} &= -\frac{\partial \boldsymbol{\mathcal{C}}}{\partial t} - \nabla\psi \end{aligned} \tag{S11}$$

When doing computations in the Coulomb gauge, where the scalar potentials $\varphi = \psi = 0$, we can easily express the electric and magnetic field in terms of the vector potentials through:

$$E^j = i\omega A^j(\omega, r), \qquad H^j = i\omega C^j(\omega, r), \tag{S12}$$

which implies that we can express the four vector potential as:

$$A^\mu = \frac{1}{i\omega}\begin{pmatrix} 0 \\ \mathbf{E} \end{pmatrix}, \qquad C^\mu = \frac{1}{i\omega}\begin{pmatrix} 0 \\ \mathbf{H} \end{pmatrix}. \tag{S13}$$

Working in the Coulomb gauge simplifies greatly the form of the spin decomposition, giving a clearer physics intuition. However, it is possible to derive all the results presented in the main work without choosing a gauge. In a general gauge, we found that the total spin is given by:

$$\begin{aligned} S^{\mu} &= \frac{1}{4}\operatorname{Re}\left\{A_{\nu}^{*}G^{\nu\mu} + C_{\nu}^{*}F^{\nu\mu}\right\} = \frac{1}{4}\operatorname{Re}\left\{\begin{pmatrix} \frac{1}{c}\left(\mathbf{A}\cdot\mathbf{H}^{*} - \mathbf{C}\cdot\mathbf{E}^{*}\right) \\ \mu\mathbf{H}^{*}\times\mathbf{C} + \varepsilon\mathbf{E}^{*}\times\mathbf{A} \end{pmatrix} + \frac{1}{c^{2}}\begin{pmatrix} 0 \\ \phi\mathbf{H}^{*} - \psi\mathbf{E}^{*} \end{pmatrix}\right\} \\ &= \frac{1}{4\omega}\operatorname{Im}\left\{\begin{pmatrix} -\frac{2}{c}\mathbf{E}^{*}\cdot\mathbf{H} \\ \epsilon_{0}\mathbf{E}^{*}\times\mathbf{E} + \mu_{0}\mathbf{H}^{*}\times\mathbf{H} \end{pmatrix} + \begin{pmatrix} \frac{1}{c}\nabla\cdot\left(\psi\mathbf{E}^{*} - \phi\mathbf{H}^{*}\right) \\ \nabla\times\left(\varepsilon\phi\mathbf{E}^{*} + \mu\psi\mathbf{H}^{*}\right) \end{pmatrix}\right\}. \end{aligned} \tag{S14}$$

Interestingly, the extra terms for a general gauge are total derivatives, meaning that the integrated helicity is gauge invariant up to a boundary term. This is because generically, total derivatives vanish under suitable boundary conditions. The canonical and Poynting spin now read as:

$$\begin{aligned} S_{C}^{\mu} &= \frac{1}{4}\operatorname{Re}\left\{A_{\nu}^{*}\left(\partial^{\mu}C^{\nu}\right) - C_{\nu}^{*}\left(\partial^{\mu}A^{\nu}\right)\right\} = \frac{1}{4}\operatorname{Re}\left\{\begin{pmatrix} -2i\omega(\phi^{*}\psi)/c^{3} + i\omega(\mathbf{A}^{*}\cdot\mathbf{C} - \mathbf{C}^{*}\cdot\mathbf{A})/c \\ \mathbf{A}^{*}\cdot(\nabla)\mathbf{C} - \mathbf{C}^{*}\cdot(\nabla)\mathbf{A} + \frac{1}{c^{2}}(\psi^{*}\nabla\phi - \phi^{*}\nabla\psi) \end{pmatrix}\right\}, \\ S_{P}^{\mu} &= \frac{1}{4}\operatorname{Re}\left\{C_{\nu}^{*}\left(\partial^{\nu}A^{\mu}\right) - A_{\nu}^{*}\left(\partial^{\nu}C^{\mu}\right)\right\} = \frac{1}{4}\operatorname{Re}\left\{\begin{pmatrix} 2i\omega(\phi^{*}\psi)/c^{3} + \left[(\mathbf{C}\cdot\nabla)^{*}\phi - (\mathbf{A}\cdot\nabla)^{*}\psi\right]/c \\ \nabla\times(\mathbf{A}\times\mathbf{C}^{*}) - \mathbf{A}^{*}(\partial_{\mu}C^{\mu}) + \mathbf{C}^{*}(\partial_{\mu}A^{\mu}) \end{pmatrix}\right\}, \end{aligned} \tag{S15}$$

where $\partial_{\mu}A^{\mu} = -i\omega\phi/c^{2} + \nabla\cdot\mathbf{A}$ and same for C, which is quantity that is zero in both Lorentz and Coulomb gauge. An instantaneous dual symmetric gauge independent helicity current density four-vector is [8]

$$S^{\mu} = \frac{1}{2}\left(\mathcal{A}_{\nu}\mathcal{G}^{\nu\mu} + \mathcal{C}_{\nu}\mathcal{F}^{\nu\mu}\right) = \frac{1}{2}\begin{pmatrix} \frac{1}{c}\left(\boldsymbol{\mathcal{A}}\cdot\boldsymbol{\mathcal{H}} - \boldsymbol{\mathcal{C}}\cdot\boldsymbol{\mathcal{E}}\right) \\ \mu\boldsymbol{\mathcal{H}}\times\boldsymbol{\mathcal{C}} + \varepsilon\boldsymbol{\mathcal{E}}\times\boldsymbol{\mathcal{A}} \end{pmatrix} + \frac{1}{2c^{2}}\begin{pmatrix} 0 \\ \varphi\boldsymbol{\mathcal{H}} - \psi\boldsymbol{\mathcal{E}} \end{pmatrix}. \tag{S16}$$

which can be decomposed into the canonical and Poynting spins in an arbitrary gauge as follows:

$$\begin{aligned} S_{C}^{\mu} &= \frac{1}{2}\left[\mathcal{A}_{\nu}\left(\partial^{\mu}\mathcal{C}^{\nu}\right) - \mathcal{C}_{\nu}\left(\partial^{\mu}\mathcal{A}^{\nu}\right)\right] = \frac{1}{2}\begin{pmatrix} \frac{1}{c}\left(\boldsymbol{\mathcal{A}}\cdot\boldsymbol{\mathcal{H}} - \boldsymbol{\mathcal{C}}\cdot\boldsymbol{\mathcal{E}}\right) \\ \boldsymbol{\mathcal{A}}\cdot(\nabla)\boldsymbol{\mathcal{C}} - \boldsymbol{\mathcal{C}}\cdot(\nabla)\boldsymbol{\mathcal{A}} \end{pmatrix} + \frac{1}{2c}\begin{pmatrix} \mathcal{A}^{\mu}\partial_{\mu}\psi - \mathcal{C}^{\mu}\partial_{\mu}\varphi \\ \frac{1}{c}\left(\psi\nabla\varphi - \varphi\nabla\psi\right) \end{pmatrix}, \\ S_{P}^{\mu} &= \frac{1}{2}\left[\mathcal{C}_{\nu}\left(\partial^{\nu}\mathcal{A}^{\mu}\right) - \mathcal{A}_{\nu}\left(\partial^{\nu}\mathcal{C}^{\mu}\right)\right] = \frac{1}{2}\begin{pmatrix} 0 \\ (\boldsymbol{\mathcal{C}}\cdot\nabla)\boldsymbol{\mathcal{A}} - (\boldsymbol{\mathcal{A}}\cdot\nabla)\boldsymbol{\mathcal{C}} \end{pmatrix} - \frac{1}{2c}\begin{pmatrix} \mathcal{A}^{\mu}\partial_{\mu}\psi - \mathcal{C}^{\mu}\partial_{\mu}\varphi \\ \frac{1}{c}\left(\psi\nabla\varphi - \varphi\nabla\psi\right) \end{pmatrix} + \frac{1}{2c^{2}}\begin{pmatrix} 0 \\ \varphi\boldsymbol{\mathcal{H}} - \psi\boldsymbol{\mathcal{E}} \end{pmatrix}, \end{aligned} \tag{S17}$$

Note that $(\boldsymbol{\mathcal{C}}\cdot\nabla)\boldsymbol{\mathcal{A}} - (\boldsymbol{\mathcal{A}}\cdot\nabla)\boldsymbol{\mathcal{C}} = \nabla\times(\boldsymbol{\mathcal{A}}\times\boldsymbol{\mathcal{C}}) + \boldsymbol{\mathcal{C}}(\nabla\cdot\boldsymbol{\mathcal{A}}) - \boldsymbol{\mathcal{A}}(\nabla\cdot\boldsymbol{\mathcal{C}})$ and $\mathcal{A}^{\mu}\partial_{\mu}\psi = \varphi(\partial\psi/\partial t)/c^{2} + \boldsymbol{\mathcal{A}}\cdot\nabla\psi$ and similarly $\mathcal{C}^{\mu}\partial_{\mu}\varphi = \psi(\partial\varphi/\partial t)/c^{2} + \boldsymbol{\mathcal{C}}\cdot\nabla\varphi$.

References

1. Shi, P., Du, L., Li, C., Zayats, A. V. & Yuan, X. Transverse spin dynamics in structured electromagnetic guided waves. *Proceedings of the National Academy of Sciences* **118** (6 Feb. 2021).
2. Bliokh, K. Y. & Nori, F. Spin and orbital angular momenta of acoustic beams. *Physical Review B* **99** (17 May 2019).
3. Bliokh, K. Y., Punzmann, H., Xia, H., Nori, F. & Shats, M. Field theory spin and momentum in water waves. *Science Advances* **8** (3 Jan. 2022).
4. Golat, S., Lim, E. A. & Rodrıéguez-Fortuño, F. J. Evanescent Gravitational Waves. *Phys. Rev. D* **101,** 084046. arXiv: `1903.09690 [astro-ph.CO]` (2020).
5. Barnett, S. M. Maxwellian theory of gravitational waves and their mechanical properties. *New Journal of Physics* **16** (2 Feb. 2014).
6. Bliokh, K. Y. & Nori, F. Transverse spin and surface waves in acoustic metamaterials. *Physical Review B* **99** (2 Jan. 2019).
7. Golat, S. & Rigouzzo, C. *Spin Decomposition* Available at `https://github.com/crigouzzo/spin-decomposition`.
8. Cameron, R. P. & Barnett, S. M. Electric–magnetic symmetry and Noether's theorem. *New Journal of Physics* **14,** 123019 (Dec. 2012).
